# The Role of Thyroid Hormones, Vitamins, and Microelements in Female Infertility

**DOI:** 10.1055/s-0043-1772478

**Published:** 2023-11-29

**Authors:** Sveta Aghayeva, Murat Sonmezer, Yavuz Emre Şükür, Aytaj Jafarzade

**Affiliations:** 1 Department of Obstetrics and Gynecology, Koru Hospital Ankara, Ankara, Turkey; 2 Department of Obstetrics and Gynecology, Ankara University, Ankara, Turkey

**Keywords:** infertility, vitamin B12, vitamin D, folic acid, ferritin, zinc, thyroid hormones, infertilidade, vitamina b12, vitamina D, ácido fólico, ferritina, zinco, hormônios da tireoide

## Abstract

**Objective** It is well known that female infertility is multifactorial. Therefore, we aimed to compare the effects of thyroid dysfunction, vitamin deficiency, and microelement deficiency in fertile and infertile patients.

**Materials and Methods** Between May 1st, 2017, and April 1st, 2019, we conducted a retrospective case-control study with of 380 infertile and 346 pregnant patients (who normally fertile and able to conceive spontaneously). The fertile patients were selected among those who got pregnant spontaneously without treatment, had a term birth, and did not have systemic or obstetric diseases. The levels of thyroid-stimulating hormone (TSH), triiodothyronine (T3), thyroxine (T4), anti-thyroid peroxidase (anti-TPO), vitamin D, vitamin B12, folic acid, ferritin, and zinc of both groups were compared.

**Results** There was no difference between patients in the infertile and pregnant groups in terms of low normal and high serum T3 and T4 levels ( *p* = 0.938; *p* > 0.05) respectively, nor in terms of normal and high anti-TPO levels ( *p* = 0.182; *p* > 0.05) respectively. There was no significant difference regarding patients with low, insufficient, and sufficient vitamin D levels in the infertile and pregnant groups ( *p* = 0.160; *p* >0.05) respectively. The levels of folic acid, ferritin, and zinc of the infertile group were significantly lower than those of the pregnant group.

**Conclusion** The serum levels of folic acid, ferritin, and zinc in infertile patients presenting to our outpatient clinic were lower than those o the fertile patients.

## Resumo

**Objetivo** Sabe-se que a infertilidade feminina é multifatorial. Portanto, nosso objetivo foi comparar os efeitos da disfunção tireoidiana, deficiência de vitaminas e deficiência de microelementos em pacientes férteis e inférteis.

**Materiais e Métodos** Entre 1° de maio de 2017 e 1° de abril de 2019, realizamos um estudo retrospectivo caso-controle com 380 pacientes inférteis e 346 grávidas (normalmente férteis e capazes de conceber espontaneamente). As pacientes férteis foram selecionadas entre aquelas que engravidaram espontaneamente sem tratamento, tiveram parto a termo e não apresentavam doenças sistêmicas ou obstétricas. Os níveis de hormônio estimulante da tireoide (TSH), triiodotironina (T3), tiroxina (T4), antitireoide peroxidase (anti-TPO), vitamina D, vitamina B12, ácido fólico, ferritina e zinco de ambos os grupos foram comparados.

**Resultados** Não houve diferença entre as pacientes dos grupos inférteis e gestantes em relação aos níveis altos de sérumT3 e T4 normais baixos e altos (p = 0,938; p > 0,05), respectivamente nem aos níveis normais e altos de anti-TPO (p = 0,182; p > 0,05), respectivamente. Não houve diferença significativa em relação aos pacientes com níveis baixos, insuficientes e suficientes de vitamina D nos grupos inférteis e gestantes (p = 0,160; p > 0,05), respectivamente. Os níveis de ácido fólico, ferritina e zinco do grupo infértil foram significativamente menores do que os do grupo grávida.

**Conclusão** Os níveis de sérum de ácido fólico, ferritina e zinco nas pacientes inférteis atendidas em nosso ambulatório foram menores do que nas pacientes férteis.

## Introduction

Infertility is a public health problem affecting 15% of couples of childbearing age, 1 and it has a negative impact on psychological well-being in both developed and underdeveloped societies. Studies 2 have shown that the level of psychological stress of infertile couples is comparable to that of patients with a cancer diagnosis or heart disease. It is known that the male factor of cases ranges from 20% to 35%, the female factor, also from 20% to 35%, and the unexplained group, from 10% to 20%. 3

Many factors can influence female infertility, and one of the most important is undiagnosed and untreated thyroid disease. In primary hypothyroidism, high levels of thyrotropin-releasing hormone (TRH) are secreted to increase the levels of thyroid-stimulating hormone (THS), resulting in hyperprolactinemia, oligomenorrhea, and anovulation. 4 Hypothyroidism can cause miscarriage, premature birth, and neurodevelopmental disorders. 5 Thyroid antibodies spontaneously lead to negative results in in vitro fertilization (IVF) because their molecular mimicry interferes with the interaction between the zona pellucida and the sperm cell. 6 Therefore, intracytoplasmic sperm injection (ICSI) is preferred in women with positive thyroid autoantibodies. 7 Moreover, thyroid autoantibodies negatively affect the embryo and lead to early pregnancy loss. 8 It is known that TSH levels in euthyroid patients do not affect the results of intrauterine insemination. 9

Vitamin B12, as a cofactor, can normalize high levels of homocysteine by converting homocysteine into methionine with the help of the enzyme folic acid methionine synthetase. 10 High homocysteine levels, which occur in patients with folic acid and B12 deficiency, impair oocyte maturation and embryo quality. 11 In patients with normal blood cobalamin and folic acid levels, it results in low homocysteine levels and, thus, better embryo quality. 12

Vitamin D plays a role in the expression of the *HOXA-10* gene in endometrial stromal cells; it also regulates immune responses during implantation, and may lead to implantation failure or embryo immune rejection if deficient. 13 14 In addition, vitamin D has been shown to increase the expression of 3b-hydroxysteroid dehydrogenase and progesterone production by inhibiting follicle-stimulating hormone (FSH) and anti-Müllerian hormone (AMH) gene receptors in granulosa cells. 15 Therefore, studies 16 advocate keeping serum vitamin D concentrations within normal limits in infertile patients.

Zinc is well known to be a cofactor of ∼ 200 enzymes, and it regulates DNA replication and meiosis. 17 When zinc is deficient, meiotic division occurs in oocytes at metaphase 2. 18 In addition, a relationship between zinc and low birth weight has been demonstrated. 19

There are studies 20 advocating that unexplained infertility is associated with low ferritin levels, that it leads to recurrent miscarriage in infertile patients, that it should become routine in infertile patients, and that substitution should be performed in infertile cases before treatment.

In light of this information, we aimed to investigate the relationship involving the serum levels of TSH, triiodothyronine (T3), thyroxine (T4), vitamin B12, folic acid, zinc, vitamin D, and ferritin in infertile patients and infertility.

## Materials and Methods

Between May 1st, 2017 and April 1st, 2019, in the Department of Gynecology and Obstetrics, Ankara University, we conducted a retrospective case-control study with pregnant and infertile patients aged between 18 and 40 years. The infertile group was composed of 380 patients who had presented to the reproductive health outpatient clinic for the first time (and had not been previously treated for infertility), and the pregnant group was composed of 346 patients who were followed up in the clinic, had given birth spontaneously, had not received any treatment or medical support, had had no obstetric or systemic problems during their pregnancy, and whose first-trimester blood parameters could be determined. The pregnant group was accepted as the fertile group, and their blood results from weeks 7 to10 were obtained and studied. The exclusion criteria for the infertile patients were: severe oligospermia or azoospermia, uterine abnormalities (septate, unicornuate, bicornuate, and didelphis uterus, and others), and submucosal myoma or endometrial polyps. The hysterosalpingography of all infertile patients included was examined, and those with tubal junction anomalies and hydrosalpinx were excluded.

In the pregnant group, we included patients whose blood results from weeks 7 to 10 of gestation were available. In both groups, we selected patients who had not taken any vitamin or food supplements in the previous six months. The exclusion criteria for the pregnant group were patients with a diagnosis of infertility and pregnancy for as treatment, patients diagnosed with habitual abortion, patients with a history of thrombophilia, patients with endocrine, metabolic, hematologic, or genetic diseases, epilepsy patients, those who had received high-dose folic acid therapy due to an abnormality in the previous pregnancy, and those who had taken vitamin or food supplements in the previous six months.

The groups were compared based on serum levels of TSH, T3, T4, anti-thyroid peroxidase (anti-TPO), vitamin D, B12, folic acid, ferritin, and zinc.

We defined infertile couples as those for whom pregnancy had not occurred despite regular sexual intercourse for 1 year if the woman was younger than 35 years and for 6 months if the woman was older than 35 years.

We obtained written approval from the institutional Ethics Committee (number I-4–149–19).

The data collected for the statistical analysis were recorded using the IBM SPSS Statistics for Windows software (IBM Corp., Armonk, NY, United States). The Shapiro-Wilk normality test was used to evaluate the distribution of the data. In line with the results of this test, the Student *t* -test or the Mann-Whitney U test were used to compare the continuous variables, and the Chi-squared test was used to assess the distribution of the categorical variables.

## Results

We included a total of 726 patients, 380 in the infertile group and 346 in the pregnant group. The mean age of the infertile group was of 30.57 years ( *p* = 0.000), and that of the pregnant group was o 28 years ( *p* < 0.001). In the infertile group, 67 (17.9%) patients had secondary infertility, and 308 (82.1%) had primary infertility. There was no difference between subjects in the infertile and fertile groups in terms of smoking status ( *p* = 0.088; *p* > 0.05 respectively). The body mass index (BMI) of the infertile group was significantly higher than that of the pregnant group ( *p* = 0.001) ( Table 1 ).

**Table 1:** Characteristics of the study sample

	Infertile group (n = 380)	Pregnant group (n = 346)	*p* -value
Mean age (years)	30,57 ± 6,503	28,07 ± 5,189	< 0.001
Mean body mass index (kg/m ^2^ )	26.227 ± 4.38086	25.0476 ± 4.48651	0.001
Smoker: n (%)	75 (20.7)	47(15.6)	0.088

The standard reference range was of 1.0 nmol/mL to 2.9 nmol/mL for T3, of 50 nmol/mL to 150 nmol/L for T4, and > 30 IU/mL for anti-TPO positivity. According to TSH levels, the patients were divided into 3 subgroups: < 2.5 mIU/mL, 2.5–4.5 mIU/mL, and > 4.5 mIU/mL. There was no difference between patients in the infertile and pregnant groups in terms of low normal and high serum T3 and T4 levels ( *p* = 0.938; *p* > 0.05) respectively. A significant difference was found between pregnant and infertile patients in all TSH level subgroups: while there were more patients with TSH < 2.5 mIU/mL in the pregnant group, a higher proportion of patients with TSH levels between 2.5 mIU/mL and 4.5 mIU/mL and > 4.5 mIU/mL were found in the infertile group. Subclinical hypothyroidism was found in ∼ 3.3% of patients in the pregnant group and in ∼ 13% of patients in the infertile group. There was no difference between the pregnant and infertile groups in terms of normal and high anti-TPO levels ( *p* = 0.182; *p* > 0.05) respectively. In total, 95.8% of patients in the pregnant serum T3 group, 96.1% of patients in the infertile group, and in the pregnant serum T4 group, 99.7% of patients were found to be normal in 98.3% of patients in the infertile group ( Table 2 ).

**Table 2:** Comparison of the results of the thyroid function test between infertile and pregnant patients

		Infertile group: n (%)	Pregnant group: n (%)	P- *value*
T3	Low	1 (0.3)	2 (0.6)	> 0.05
	Normal	345 (96.1)	321 (95.8)	
	High	13 (3.6)	12 (3.6)	
T4	Low	1(%0.3)	0	> 0.05
	Normal	354 (98.3)	339 (99.7)	
	High	5 (1.4)	1 (0.3)	
TSH	< 2.5 mIU/L	221 (59.7)	296 (87.3)	< 0.001
	2.5–4.5mIU/L	101 (27.3)	32 (9.3)	
	> 4.5 mIU/L	48(%13)	11(%3.3)	
Anti-TPO	Normal	266 (82.7)	256 (86.5)	> 0.05

Abbreviations: Anti-TPO, anti-thyroid peroxidase; T3, triiodothyronine; T4, thyroxine; TSH, thyroid-stimulating hormone.

In line with many previous studies, in the present study, we classified vitamin D levels > 30 ng/mL as adequate, levels between 20 30 ng/mL and 30 ng/ml, as inadequate, and levels < 20 ng/mL as low. There was no significant difference regarding patients with low, insufficient, and sufficient levels of vitamin D in the infertile and pregnant groups ( Table 3 ) ( *p* = 0.160; *p* > 0.05 respectively). Vitamin D levels were considered sufficient in 5.4% of the patients in the infertile group and in 8.9% of the patients in the pregnant patient group. Considering the whole sample, vitamin D levels were sufficient in 7.1% of the patients.

**Table 3:** Comparison of vitamin D values

Group	Vitamin D			Total
	Low	Insufficient	Sufficient	
Infertile group: n (%)	255 (76.1)	62 (18.5)	18 (5.4)	335 (100)
	Pregnant group: n (%)	239 (75.6)	49 (15.5)	28 (8.9)	316 (100)
Total: n (%)		494 (75.9)	111 (17.1)	46 (7.1%)	651 (100)

Low vitamin D levels were found in 76.1% of the infertile group and in 75.6% of the pregnant group. Deficiency was defined as following serum levels: vitamin B12 < 200 pg/mL; folic acid < 5.9 ng/mL; zinc < 76 mg/mL; and ferritin < 11ng/mL. Serum vitamin B12 levels were found in 17.7% of the infertile group and in 21.5% of the pregnant group, with no significant difference between the groups. In the infertile group, the levels of folic acid, ferritin, and zinc were significantly lower than those of the pregnant group. Miscarriage was observed in 26 patients (0.78%) of 333 the patients in the folic acid pregnant group and in 95 (0.25%) of the 366 patients in the infertile group. Ferritin levels were low in 155 (45.5%) out of 341 infertile patients and in 98 (29.2%) out of 336 pregnant women. The levels of zinc were assessed in 130 pregnant women and in 163 infertile patients, and it was low in 13.1% of the pregnant group and in 23.9% of the infertile group ( Table 4 ).

**Table 4:** Comparison of the levels of vitamin B12, folic acid, ferritin, and zinc in the infertile and pregnant groups

		Infertile group: n (%)	Total sayı: n	Pregnant group: n (%)	Total sayı: n	*p* -value
Vitamin B12	Low	66 (17.7)	372	73 (21.5)	339	> 0.05
	Normal	306 (82.3)		266 (78.5)		
Folic acid	Low	95 (26)	366	26 (7.8)	333	< 0.001
	Normal	271 (74)		307 (92.2)		
Ferritin	Low	155 (45.5)	341	98 (29.2)	336	< 0.001
	Normal	186 (54.5)		238 (70.8		
Zinc	Low	39 (23.9)	163	17 (13.1)	130	< 0.001
	Normal	124 (76.1)		113 (86.9)		

## Discussion

In the present study, we found no significant differences between the infertile and pregnant groups in terms of the levels of T3, T4, anti-TPO, vitamin D and vitamin B12, and the serum levels of ferritin, folic acid, and zinc were significantly lower in the infertile patient group compared with the pregnant group.

A study on the effect of vitamin D on infertility and endometriosis found that adequate vitamin D levels (≥ 30 ng/mL) should be ensured in women undergoing IVF treatment. Studies 21 show that vitamin D supplementation regulates serum lipid levels in patients with polycystic ovary syndrome, and it reduces the risk of endometriosis.

In another study, 21 although the authors noted that vitamin D and its metabolites play an important role in embryo implantation and immunologic protection of the embryo and that couples with serum vitamin D concentrations higher than 50 nmol/L have a higher chance of becoming pregnant, they stated that this is not true for all patients. 22 The reason why this study 22 does not support the results of the present study might be because both studies were conducted with different populations. Studies 23 have shown that vitamin D deficiency (< 10 ng/mL) is also associated with the presence of thyroid antibodies, and that TSH levels tend to have a direct relationship with vitamin D status in women with thyroid autoimmunity as opposed to those without.

In the present study, although the rate of patients with serum TSH levels < 2.5 mIU/mL was significantly lower in the infertile group compared with the pregnant group, no significant relationship was found between both groups in terms of the levels of T3, T4, and anti-TPO. Similar to the results of the present study, in a large retrospective study 24 with 11,254 patients from Denmark, the authors found that infertility and subclinical hypothyroidism were associated. Another study 25 stated that the levels of T3 and T4 were not associated with conception; however, anti-TPO might reduce the quality of the oocyte. In the present study, the rate of patients with TSH levels between 2.5 mIU/mL and 4.5 mIU/mL and > 4.5 mIU/mL was higher in the infertile group, which is in line with previous studies 26 investigating the same parameters in infertile patients. In addition, the prevalence of thyroid autoimmune diseases was higher in women with polycystic ovary syndrome and idiopathic subfertility. Therefore, women with hypothyroidism should be treated until their serum levels of TSH reach < 2.5 mIU/mL before undergoing therapy with assisted reproductive techniques (ARTs). Euthyroidism should be restored and maintained several months before starting the ART therapy. Fertilization rates and embryo quality may deteriorate in women with TSH > 4.0 mIU/mL. 26 Meta-analysis studies 26 mainly including women with TSH levels > 4.0 mIU/mL have shown that the rates of live birth increase after hypothyroidism treatment. However, autoimmune thyroid disease with euthyroidism increases the live birth rate in women with the disease. 27

Similarly, in a study published in 2017 by Irene La Vecchia et al., 28 the levels of vitamin A, vitamin E, folic acid, vitamin B12, and ferritin were investigated among infertile patients. This study 28 differed from ours in the sense that the infertile patients were not compared with fertile patients. Vitamin B12 levels were low in 66% of 269 patients, and folic acid levels were low in 22% 28 In the present study, low levels of vitamin B 12 were detected in 17.7% of the infertile and 21.5% of the pregnant group. Folic acid was observed to be low in 26% of the infertile group, similar to the result found by La Vecchia et al. 28 Ferritin levels were adequate in more than 80% of patients assessed by La Vecchia et al. 28 In the present study, we observed low levels of ferritin r45.5% of the infertile patients. In similar studies, 29 the levels of folic acid increased the success of the ART treatment by decreasing serum homocysteine levels, especially in patients with unexplained infertility. High serum levels of vitamin D and folic acid increase oocyte quality and the release of follicular estradiol, thus positively affecting fertility. 30 Another study 31 stated that IVF success was higher because more metaphase II (MII) oocytes were obtained from patients who took vitamin B12 supplements. In addition, low serum levels of ferritin were associated with recurrent pregnancy loss. 32 A study 33 conducted at an infertility center in the United States revealed that normal plasma antioxidant (zinc, selenium, and vitamin E) levels within normal reference ranges do not benefit male fertility. Studies 34 32 on ferritin and zinc in infertility have generally focused on male infertility. However, in the present study, we found a relationship involving serum levels of zinc and ferritin and female infertility. Nevertheless, there is a need for prospective multicentric studies with larger samples. In a study with polycystic ovarian patients, 34 the authors found no correlation between the serum levels of zinc in the case and control groups In addition, another study 32 found that ferritin levels were significantly lower in patients with recurrent pregnancy loss compared with the control group. 32

Although the present study is not on a new subject, it can be considered an extension of previous studies with large samples that investigated the association of vitamin D, vitamin B12, folic acid, ferritin, zinc, and thyroid hormones with infertility. In addition, the fact that the present study was conducted in a different population may have caused our data to differ from previously reported data. The sample size of the present study is sufficient to identify vitamin D, B12, folic acid, ferritin, zinc, and thyroid hormones in infertility. We only included white patients to ensure the study's homogeneity. Still, in terms of mean age, the infertile group was older than the pregnant group. A limitation of the present study is that it did not consider the lifestyles, eating habits, socioeconomic status, or practice of physical activities of the patients. Thus, there may be differences in certain serum blood levels, especially in those of vitamins and microelements.

## Conclusion

In the present study, we found that the serum levels of vitamin D and vitamin B12 of the infertile patients were as low as those of fertile patients. The levels of folic acid, ferritin, and zinc were also lower in infertile patients. In addition, subclinical hypothyroidism was observed more frequently in infertile patients. Prospective studies with larger samples are needed.
